# An Internet-Based Epidemiological Investigation of the Outbreak of H7N9 Avian Influenza A in China Since Early 2013

**DOI:** 10.2196/jmir.3763

**Published:** 2014-09-25

**Authors:** Chen Mao, Xin-Yin Wu, Xiao-Hong Fu, Meng-Yang Di, Yuan-Yuan Yu, Jin-Qiu Yuan, Zu-Yao Yang, Jin-Ling Tang

**Affiliations:** ^1^School of Public Health and Primary Care, The Chinese University of Hong Kong, Hong KongHong KongChina (Hong Kong); ^2^The Shenzhen Municipal Key Laboratory for Health Risk Analysis, Shenzhen Research Institute of The Chinese University of Hong KongShenzhenChina

**Keywords:** influenza A virus, H7N9 subtype, Internet, big data, disease outbreaks, epidemiology

## Abstract

**Background:**

In early 2013, a new type of avian influenza, H7N9, emerged in China. It quickly became an issue of great public concern and a widely discussed topic on the Internet. A considerable volume of relevant information was made publicly available on the Internet through various sources.

**Objective:**

This study aimed to describe the outbreak of H7N9 in China based on data openly available on the Internet and to validate our investigation by comparing our findings with a well-conducted conventional field epidemiologic study.

**Methods:**

We searched publicly accessible Internet data on the H7N9 outbreak primarily from government and major mass media websites in China up to February 10, 2014. Two researchers independently extracted, compared, and confirmed the information of each confirmed H7N9 case using a self-designed data extraction form. We summarized the epidemiological and clinical characteristics of confirmed H7N9 cases and compared them with those from the field study.

**Results:**

According to our data updated until February 10, 2014, 334 confirmed H7N9 cases were identified. The median age was 58 years and 67.0% (219/327) were males. Cases were reported in 15 regions in China. Five family clusters were found. Of the 16.8% (56/334) of the cases with relevant data, 69.6% (39/56) reported a history of exposure to animals. Of the 1751 persons with a close contact with a confirmed case, 0.6% (11/1751) of them developed respiratory symptoms during the 7-day surveillance period. In the 97.9% (327/334) of the cases with relevant data, 21.7% (71/327) died, 20.8% (68/327) were discharged from a hospital, and 57.5% (188/327) were of uncertain status. We compared our findings before February 10, 2014 and those before December 1, 2013 with those from the conventional field study, which had the latter cutoff date of ours in data collection. Our study showed most epidemiological and clinical characteristics were similar to those in the field study, except for case fatality (71/327, 21.7% for our data before February 10; 45/138, 32.6% for our data before December 1; 47/139, 33.8% for the field study), time from illness onset to first medical care (4 days, 3 days, and 1 day), and time from illness onset to death (16.5 days, 17 days, and 21 days).

**Conclusions:**

Findings from our Internet-based investigation were similar to those from the conventional field study in most epidemiological and clinical aspects of the outbreak. Importantly, publicly available Internet data are open to any interested researchers and can thus greatly facilitate the investigation and control of such outbreaks. With improved efforts for Internet data provision, Internet-based investigation has a great potential to become a quick, economical, novel approach to investigating sudden issues of great public concern that involve a relatively small number of cases like this H7N9 outbreak.

##  Introduction

The modern world is becoming increasingly connected by the Internet, which comprises a large system of various private, public, business, academic, and government networks that facilitate rapid and open data exchange among billions of people worldwide [1]. The Internet has removed a major constraint to accessing and sharing data, information, and knowledge. Unlike traditional media, it provides an open platform that allows various people to report, confirm, and correct details regarding issues of public concern. For example, “Internet mass hunting,” which literally means to uncover the true identity of a particular person or matter through the coordinated efforts of all “netizens,” is an approach to using the Internet for conducting investigations [2]. In China, this approach has been increasingly employed by the public to elucidate issues of public concern, such as cases of corruption [3].

The Internet also provides novel opportunities for health research. Eysenbach developed a method for analyzing Google queries to track cases of influenza-type illnesses in a given population [4,5]. That method was employed to accurately predict weekly influenza activity in each region in the United States with a reporting lag of approximately 1 day [6]. Health surveys and clinical trials have also been conducted through the Internet [7-10]. Other examples of Internet-based research in the health sciences include Infovigil, HealthMap, MedISys, BioCaster, and EpiSPIDER [11-15]. Compared with traditional field research methods, such as face-to-face interviews and paper-based questionnaires, Internet-based methods involve considerably fewer resources, including money, time, and human resources. The Internet is particularly useful for obtaining information and influencing behavioral responses during public crises [16], such as during outbreaks of new infectious diseases.

In early 2013, a new type of avian influenza, H7N9, emerged in China, becoming a matter of strong public concern. After the first case was reported, H7N9 rapidly became a widely discussed topic on the Internet. A considerable volume of relevant information was made publicly available on the Internet through various channels, including news reports, discussion forums, personal blogs, and reports from hospitals and government authorities. In this study, we aimed to describe the outbreak of H7N9 in China based on data available on the Internet by February 10, 2014, and to explore the methods, feasibility, validity, advantages, and limitations of employing the Internet-based approach to investigating public health issues by comparing our findings with those presented in a well-conducted conventional field epidemiologic study on the H7N9 outbreak [17].

## Methods

### Study Structure and Definitions

We collected publicly available Internet data related to the H7N9 outbreak from reliable websites. The field epidemiologic study by Li and colleagues [17] was based on the data they reported to the National Center for Disease Control and Prevention of the country (China CDC) and was employed as a reference for validating our study. In our study, we first summarized the epidemiological and clinical characteristics of H7N9 cases based on the data from the Internet updated until February 10, 2014. To validate our study, we compared our findings with those in Li’s study [17]. Li’s results were compared with ours based on data with the same cutoff date of investigation (December 1, 2013) and with our results updated to February 10, 2014.

In Li’s study, suspected and confirmed cases of H7N9 virus infection were defined according to the definition of H5N1 cases recommended by the World Health Organization (WHO) in 2006. Suspected cases were identified through China’s surveillance system for cases of pneumonia of unexplained origin. If laboratory tests, such as real-time reverse transcriptase–polymerase chain reaction assay, viral isolation, and serological testing, indicated the presence of the H7N9 virus, the case was considered confirmed. Once a suspected case was identified, initial field investigations were conducted and respiratory specimens were obtained by local CDCs. Information on each confirmed case was collected through field investigation until December 1, 2013.

In our study, confirmed H7N9 cases were those that claimed to be newly identified H7N9 cases reported on either government or nongovernment websites, and verified either by laboratory tests or nationally or provincially organized specialists.

### Sources of Internet Data

Various H7N9-related data were reported through numerous Internet-based resources. The data in this study were obtained from only 2 website categories: government and major media websites. These categories are detailed as follows, in the order of their trustworthiness:

Websites of government organizations providing H7N9-related information in China were the primary source of data. The 5 most representative government organizations that provided our needed information were (1) municipal health bureaus; (2) national, provincial, and prefectural CDCs; (3) the National Health and Family Planning Commission; (4) the Ministry of Agriculture; and (5) the WHO.

Social media websites were used as supplementary sources of data. Websites that set up a special column or discussion forum for the outbreak of H7N9 Avian Influenza A were given a higher priority than others. In fact, most of our supplementary data were obtained from the 2 most popular public websites in the country: Sina and Sohu. Both had a special section for H7N9 on their websites [18,19]. They provided numerous information, including live reports of new H7N9 cases, individual data for confirmed H7N9 cases, simple summary data of the outbreak, prevention and treatment of H7N9 Avian Influenza A, and comments from netizens.

Governmental data were considered most trustworthy and contributed most of the data in this study. Information was sought from social media websites when some data were missing from government websites.

We included 2 cases reported on Health Authority websites in Hong Kong, but did not search Taiwan-based websites and thus the few cases in Taiwan were not included in this study. No case was reported in Macau.

### Case Identification and Data Collection

To avoid relying on or repeating the summary or aggregate results of official reports and other epidemiological studies by those who had access to official individual data on H7N9 cases, we searched and extracted only those data on individual cases from websites with free public access. These data were mainly included in daily reports published on relevant government authority websites. Websites were selected according to usefulness, accessibility, and credibility. Usefulness was determined by the comprehensiveness and up-to-datedness of the information. To obtain data for each confirmed H7N9 case, we searched daily reports published on the CDC and other health bureau websites at the national, provincial, and prefectural level from March 31, 2013 (the date of the first reported case) to February 10, 2014. Complementary searches of social media websites, predominantly Sina and Sohu, were also performed to supplement data obtained from the government websites. Cases were identified based on the date of illness onset, family name, demographic data (gender, age, and region), and exposure history. Duplicate cases were defined as those with an identical date of illness onset, family name, gender, age, and region. When cases were reported on multiple websites, we used the data from government websites of the highest level from national to provincial to prefectural.

### Data Extraction and Quality Control

Two researchers (MYD and YYY) extracted the data for each confirmed case independently by using a self-designed data extraction form. Discrepancies were resolved by double-checking the websites and discussions if deemed necessary. Extracted data included (1) demographic data, such as age, sex, rural/urban residency, and occupation; (2) epidemiological data, such as potential exposure history to the H7N9 virus, the number of close contacts, secondary cases, familial aggregation cases, and confirming method for diagnosis; and (3) clinical data, such as hospitalization, intensive care unit (ICU) admission, development of acute respiratory distress syndrome (ARDS), death, and dates for these clinical outcomes (see Multimedia Appendix 1).

###  Statistical Analysis

We employed descriptive and analytic statistics to summarize the epidemiological and clinical characteristics of confirmed H7N9 cases. SPSS version 18.0 (SPSS Inc, Chicago, IL, USA) was used to perform all the statistical analyses.

## Results

### Epidemiologic Characteristics of Confirmed Cases by February 10, 2014

**Figure 1:**
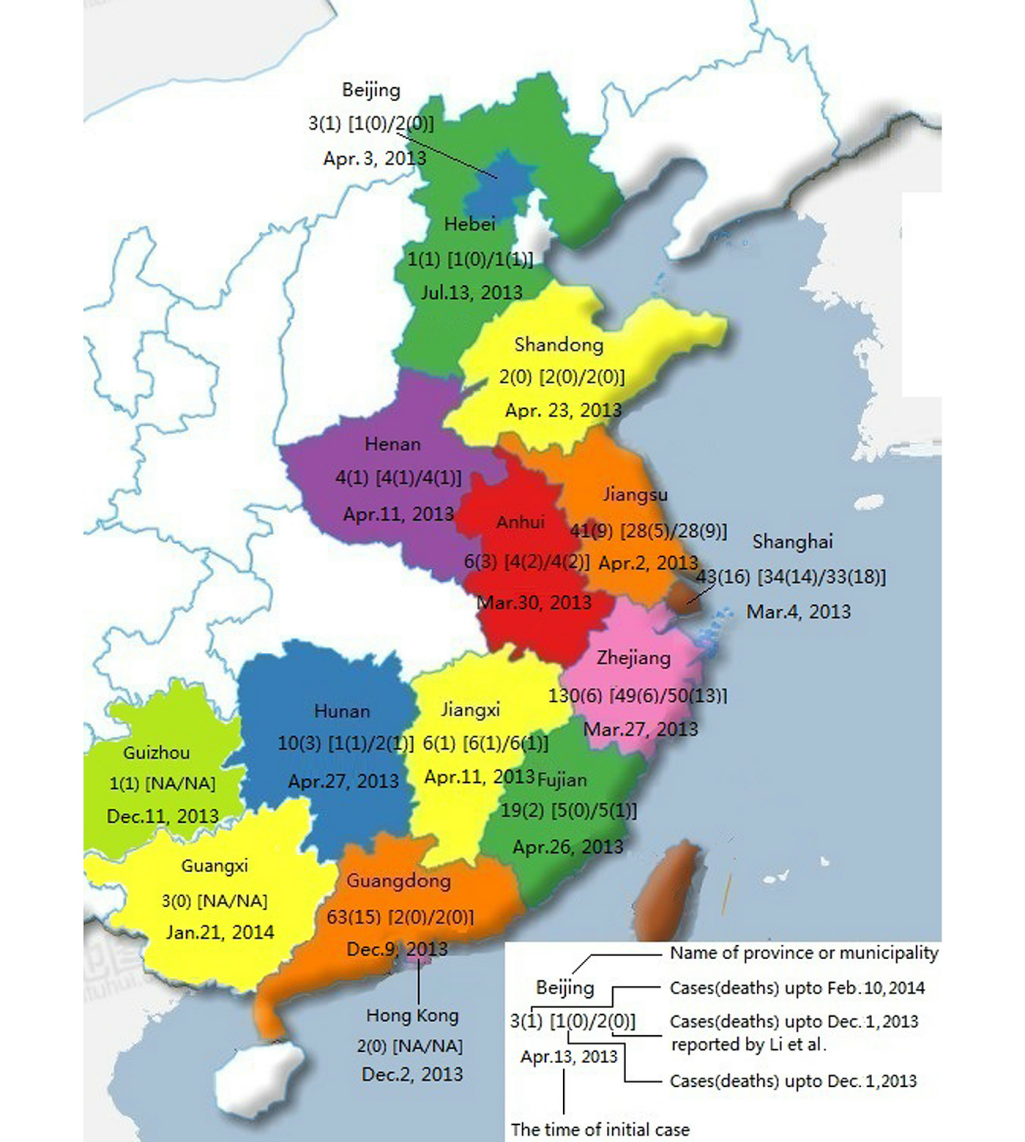
Geographic distribution of patients with confirmed H7N9 infection in China from early 2013 to February 10, 2014.

From February 17, 2013, to February 10, 2014, a total of 334 cases of H7N9 infection were identified. Cases occurred in the following 15 regions (Figure 1): (1) Zhejiang (130 cases); (2) Guangdong (62 cases); (3) Shanghai (42 cases); (4) Jiangsu (42 cases); (5) Fujian (20 cases); (6) Hunan (10 cases); (7i) Jiangxi (6 cases); (8) Anhui (6 cases); (9) Henan (4 cases); (10) Beijing (3 cases); (11) Guangxi (3 cases); (12) Shandong (2 cases); (13) Hong Kong (2 cases); (14) Hebei (1 case); and (15) Guizhou (1 case).

The majority of cases were obtained from official sources, with 89.5% (299/334) from government websites, 4.5% (15/334) from nongovernment websites, and 6.0% (20/334) from both government and nongovernment websites. Table 1 summarizes the epidemiologic characteristics of the confirmed cases. The median age was 58 years, with 33.9% (111/327) older than 65 years and 1.8% (6/327) younger than 5 years. More men (219/327, 67.0%) than women were reported as confirmed cases of H7N9. Most of the cases (186/278, 66.9%) lived in urban areas. Seven cases (7/201, 3.5%) were poultry workers. Five cases had comorbidities including hypertension, heart disease, diabetes mellitus, and chronic bronchitis. Among the 91 confirmed H7N9 cases with information on the method of diagnosis, 78.0% (71/91) were confirmed using nucleic acid detection and the remaining 20 cases were confirmed by a group of infectious disease specialists.

Information on recent exposure to animals was available for 56 of 334 confirmed cases of H7N9, 39 of which were patients with a history of exposure to animals. Cases with an animal exposure are detailed as follows: (1) 3 patients reported 1 instance of exposure to poultry; (2) 10 patients reported multiple instances of exposure to poultry; (3) 3 patients were exposed to pigeons, quails, and pet birds; and (4) the remaining 23 cases did not report the type of animals to which patients were exposed.

**Table 1:** Epidemiologic characteristics of patients with confirmed H7N9 infection in China from early 2013 to February 10, 2014.

Characteristics		Confirmed H7N9 cases^a^		
		Internet-based data (up to Dec 1, 2013)	Li’s report (up to Dec 1, 2013)	Most updated Internet-based data (up to Feb 10, 2014)
Total number of cases		138	139	334
**Age (years)**				
	Number of cases	132	139	327
	Median (IQR^b^)	61 (48-73)	61 (46-73)	58 (41-69)
	<5 years, n/N (%)	3/132 (2.3)	4/139 (2.9)	6/327 (1.8)
	≥ 65 years, n/N (%)	55/132 (41.7)	58/139 (41.7)	111/327 (33.9)
Male sex, n/N (%)		93/132 (70.5)	98/139(70.5)	219/327 (67.0)
**Area of residence, n/N (%)**				
	Urban	83/110 (75.5)	101/139 (72.6)	186/278 (66.9)
	Rural	27/110 (24.5)	38/139 (27.3)	92/278 (33.1)
Poultry worker, n/N (%)		6/99 (6.1)	9/139 (6.6)	7/201 (3.5)
Presence of underlying medical conditions, n/N (%)		3/3 (100.0)	79/108 (73.1)	5/5 (100.0)
Exposure to a symptomatic case within 2 week before illness onset, n/N (%)		5/NE^c^ (NE)	5/120 (4.2)	7^d^/NE (NE)
**Exposure to poultry, n/N (%)**		12/26 (46.0)	107/131 (81.7)	39/56 (69.6)
	Chickens	7/12 (58.3)	88/107 (82.2)	12/39 (30.8)
	Ducks	3/12 (25.0)	24/107 (22.4)	4/39 (10.3)
	Pigeons	1/12 (8.3)	13/107 (12.1)	1/39 (2.7)
	Quails	1/12 (8.3)	2/107 (1.9)	1/39 (2.7)
	Pet birds	1/12 (8.3)	3/107 (2.8)	1/39 (2.7)
	Direct contact with poultry	9/12 (75.0)	63/107 (58.9)	17/39 (43.6)
Nucleic acid detection confirming, n/N (%)		58/78 (74.4)	89/139 (64.0)	71/91 (78.0)

^a^Unless stated otherwise, data in the table provided are the number of patients with a certain characteristics, the total number of patients having data on that characteristic and the corresponding percentage.

^b^IQR: interquartile range

^c^NE: not estimable

^d^Two cases in Zhejiang were not confirmed by February 10, 2014.

### Family Clusters

Among the 334 identified H7N9 cases, 5 family clusters were reported in 3 provinces: (1) a father (confirmed) and his 2 sons (1 confirmed and the other unconfirmed) in Shanghai; (2) a married couple, both of whom were confirmed cases in Shanghai; (3) a father and son, both of whom were confirmed in Shandong; (4) a father and son-in-law, both of whom were confirmed in Zhejiang; and (5) 3 confirmed cases in the same family in Zhejiang. In the last family cluster, the man was sick first and his daughter and wife became sick after giving care to him. All 3 family members also reported an identical history of exposure to poultry. The investigation for these cases was still going on as of February 10, 2014.

### Clinical Characteristics

Table 2 summarizes the clinical characteristics and medical care timelines for confirmed cases. A total of 209 (209/223, 93.7%) cases were hospitalized, 33 (33/61, 54.1%) were admitted to ICUs, and 16 (16/23, 69.7%) developed ARDS. A total of 68 patients (68/327, 20.8%) were discharged from the hospital, 71 (71/327, 21.7%) died, and 188 (188/327, 57.5%) were of uncertain status. The median time from illness onset until their first medical visit, time from illness onset to hospitalization, time from illness onset to ICU admission, time from illness onset to the development of ARDS, and time from illness onset to death was 4.0 days (71 cases), 5.0 days (55 cases), and 6.5 days (6 cases), 6.0 days (3 cases), and 16.5 days (34 cases), respectively.

We conducted a subgroup analysis to compare the case fatality rate of confirmed H7N9 cases before and after the first reported cases on March 31, 2013. Of the 29 cases reported before March 31, 2013, 44.8% (13/29) died, and the case fatality was significantly higher (χ^2^_1_= 15.1, *P*<.001) than that in patients reported after March 31, 2013 (58/305, 19.0%).

**Table 2:** Clinical characteristics and medical care timelines for patients with confirmed H7N9 infection in China from early 2013 to February 10, 2014.

Variables		Confirmed H7N9 cases^a^		
		Internet-based data (up to Dec 1, 2013)	Li’s report (up to Dec 1, 2013)	Most updated Internet-based data (up to Feb 10, 2014)
**Clinical outcome, n/N (%)**				
	Hospitalization	118/132 (89.4)	137/139 (98.7)	209/223 (93.7)
	ICU admission	30/56 (53.6)	65/103 (63.1)	33/61 (54.1)
	ARDS^b^	14/18 (77.8)	48/83 (57.8)	16/23 (69.7)
	Death	45^d^/138 (32.6)	47/139 (33.8)	71/327 (21.7)
**Time from onset to first medical care-days**				
	Number of cases	53	137	71
	Median (IQR^c^)	3.0 (1.0-5.0)	1.0 (0-3.0)	4.0 (1.0-6.0)
**Time from onset to hospitalization-days**				
	Number of cases	41	137	55
	Median (IQR)	5.0 (3.0-6.5)	4.0 (3.0-6.0)	5.0 (3.0-6.0)
**Time from onset to ICU admission-days**				
	Number of cases	4	103	6
	Median (IQR)	7.5 (5.5-10.3)	7.0 (5.0-9.0)	6.5 (4.8-8.8)
**Time from onset to development of ARDS-days**				
	Number of cases	3	83	3
	Median (IQR)	6.0 (NE)^e^	7.0 (5.0-9.0)	6.0 (NE)^e^
**Time from onset to death-days**				
	Number of cases	25	47	34
	Median (IQR)	17.0 (10.5-22.5)	21.0 (12.5-36.0)	16.5 (9.5-22.0)

^a^Unless stated otherwise, data in the table provided are the number of patients with a certain characteristic, the total number of patients having data on that characteristic, and the corresponding percentage.

^b^ARDS: respiratory distress syndrome

^c^IQR: interquartile range

^d^This number from aggregate data on the website of National Health and Family Planning Commission. If we use individual data, only 33 deaths were found, with a case fatality rate of 23.9%.

^e^NE: not estimable

### Close Contact

By February 10, 2014, 81 confirmed H7N9 cases provided data on people who had close contact with them, with a total number of 1751 people involved. Among them, 859 occurred in Jiangsu, 310 in Shanghai, 113 in Zhejiang, 72 in Anhui, 45 in Henan, 42 in Fujian, 54 in Jiangxi, 9 in Shandong, 37 in Beijing, and 210 in Guangdong. Only 11 of these people developed respiratory symptoms during the 7-day surveillance period. Family clusters were not included in these analyses.

### Comparison With Li’s Study

By February 10, 2014, the number of cases collected from the Internet was twice more than that reported in Li’s study. By using the same cutoff date of December 1, 2013, we obtained 138 cases, which was only 1 fewer than the number of cases (139 cases) included in Li’s study.

Based on the data by February 10, 2014, and by December 1, 2013, our findings agreed with Li’s report on most epidemiological characteristics, including age, sex ratio, area of residence, and the most commonly used method for confirming cases of H7N9, although we had less detailed information on patient exposure history and clinical characteristics. Despite the problem of missing data, our results were similar to those reported in Li’s study in most clinical characteristics, such as median time from illness onset to hospitalization, ICU admission, and development of ARDS.

Based on the most updated data up to February 10, 2014, we obtained a case fatality (71/327, 21.7%) lower than that reported in Li’s study (47/139, 33.8%). If using individual data from the Internet with the same cutoff date of December 1, 2013, the case fatality in our study was 23.9% (33/138), which was still lower than 33.8% (47/139) reported in Li’s study. However, the website of the National Health and Family Planning Commission reported a total of 45 deaths by October 31, 2013. Based on this publically available aggregate data, a case fatality was very similar to that in Li’s study. Our results on both time from illness onset to first medical care and that from illness onset to death differed from those reported by Li for both cutoff dates used. We were unable to collect detailed information on the identified family clusters (Tables 1 and 2).

**Figure 2:**
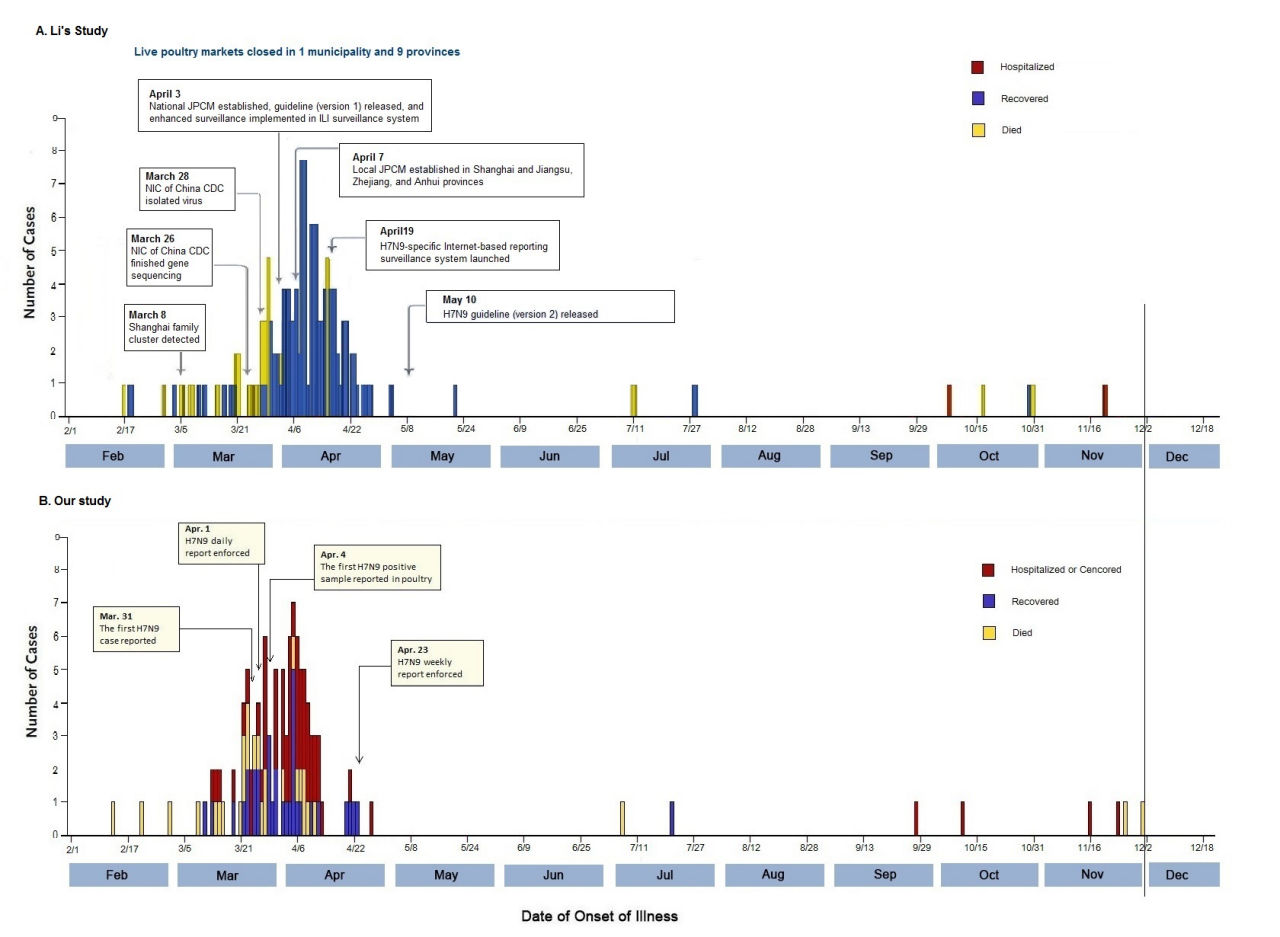
Dates of onset of illness in patients with confirmed H7N9 infection in China from early 2013 to February 10, 2014.

Based on the Internet data by December 1, 2013, the epidemic curve shown in Figure 2 is very similar to that in Li’s report, although in our study only 102 cases had the information on the date of onset of illness and there were more missing cases at end of the peak outbreak period. The first case was reported on February 19, 2013, and subsequent cases were reported sporadically between February 19 and March 27, 2013. An epidemic peak was observed between March 28 and April 18, 2013. Since then, the number of cases started to decline, with only 13 new cases reported from April 19 to December 1, 2013.

The epidemic re-emerged in 2014. Because the date of illness onset was reported for few cases in 2014, we could not show the epidemic curve for the H7N9 outbreak in 2014.

###  Comparison of the Plateaus by Using the Internet-Based Data

Based on the Internet data, the plateau stages in 2013 and 2014 were from March 31 to April 28, 2013 and from January 10 to February 10, 2014, with 118 case and 176 cases reported, respectively. During the 2 plateau periods, cases in 2014 were younger and more likely to live in rural areas than those in 2013. The fatality rate in 2014 was lower than that in 2013 and the sex ratio was similar in the 2 years (Table 3).

**Table 3:** Comparison of the characteristics of patients with confirmed H7N9 infection in China between different plateau periods

Characteristics		Confirmed H7N9 cases^a^		*P*
		The first plateau (Mar 31 to Apr 28, 2013)	The second plateau (Jan 10 to Feb 10, 2014)	
Total number of cases		118	176	NE^b^
**Age (years)**				
	Number of cases	118	175	NE^b^
	Median age (IQR^c^)	61.5 (48-74)	57.0 (41-67)	.01
	% ≥ 65 years	51 (43.2)	49 (28.0)	.01
	%<64 years	67 (56.8)	126 (72.0)	.01
**Sex, n/N (%)**				
	Male	83/118 (70.3)	115/175 (65.7)	.41
	Female	35/118 (29.7)	60/175 (34.3)	
**Area of residence, n/N (%)**				
	Urban	82/104 (78.8)	72/130 (55.4)	<.001
	Rural	22/104 (21.2)	58/130 (44.6)	
Poultry workers, n/N (%)		5/88 (5.7)	2/95 (2.1)	.27
**Clinical outcomes, n/N (%)**				
	Hospitalization	107/118 (90.7)	93/93 (100.0)	NE^b^
	ICU admission	30/56 (53.6)	2/4 (50.0)	NE^b^
	ARDS^d^	12/14 (85.7)	1/5 (20.0)	NE^b^
	Death	27/118 (22.9)	23/176 (13.1)	.03

^a^Unless stated otherwise, data in the table provided are the number of patients with a certain characteristics, the total number of patients having data on that characteristic, and the corresponding percentage.

^b^NE: not estimable

^c^IQR: interquartile range

^d^ARDS: respiratory distress syndrome

## Discussion

### Principal Findings

The findings of this study show that the epidemiological and clinical characteristics for a disease outbreak like H7N9 can be investigated by using information publicly available on the Internet. We updated the outbreak up to February 10, 2014, and found a total of 334 confirmed cases distributed in 15 regions in China. This is probably the most updated report of this outbreak so far. Patients aged between 2.5 and 91 years, but the majority were old people (median age 58 years). A total of 67.0% were males, 66.9% lived in urban areas, and 93.7% of the reported cases were hospitalized. The overall case fatality was 21.7%. We found no evidence for human-to-human transmission of the infection.

### Comparison With Prior Work

As compared with a well-conducted conventional epidemiologic study [17], the findings of our study are consistent in most of the epidemiological characteristics examined but show some discrepancies primarily in clinical characteristics of patients. In particular, the discrepancies were observed in the percentage of patients hospitalized, admitted to ICUs, and diagnosed with ARDS. In addition, our study also provided fewer details than Li’s study on the history of exposure to animals and on family clusters. Importantly, the case fatality in our study was lower than that reported in Li’s study. However, this problem can be easily corrected by the summary data available on multiple Internet sources. The discrepancies may be mainly a result of incompleteness of data on the Internet. For example, by December 1, 2013, we only acquired 53 cases with adequate information on the time from illness onset to first medical care, whereas Li reported 137 cases with relevant information.

The similarity of the results between our study and Li’s report [17] indicates that Internet-based data sources can provide useful information on basic epidemiological characteristics of an outbreak like H7N9. The features and progress of an outbreak can be studied in a timely manner and reported by those who have special analytical skills, but who would not have access to necessary data conventionally. Such research can assist the general population and health workers in acquiring a timely overview of an epidemic even before official data are published or updated.

Understandably, Internet-based data may not be complete in some particular details, which are exposure history and clinical characteristics in our case. However, the incompleteness of data may not necessarily have a major impact on understanding and controlling the outbreak because they may still be able to give a restively accurate picture about some or all these factors like many other studies that are based on samples. For example, our study showed identical results on patient history of exposure to animals. On the other hand, 196 new cases were reported after Li’s study until February 10, 2014, and our study included twice as many patients as in Li’s report. Based on these data, we updated the report and showed a new attack of the infection in early 2014.

As of February 10, 2014, Internet-based data indicated that the case fatality rate of H7N9-infected patients (21.7%) was considerably lower than that of H5N1-infected patients in both China (70%) and worldwide (59%) [20,21], but higher than that (10%) of patients with (SARS) [22]. Our study and Li’s report also indicate that the H7N9 virus tends to infect people who are much older than those infected by the H5N1 virus [20].

In addition, a subgroup analysis suggested that the case fatality rate before the first H7N9 case was reported (ie, March 31, 2013) was substantially higher than that after that. It could be partially explained by the incomplete reporting of patients with mild or no symptoms before the first case was reported. Because of the lack of awareness and limitations in the surveillance and detection process at the beginning of the outbreak, it is highly probable that the actual number of H7N9-infected cases was underestimated at the early stage of the outbreak [17]. Another possible explanation could be that the surveillance, prevention, and treatment of the disease improved after more cases were reported, which helped doctors and other health workers more effectively deal with the patients than during the early stage of the outbreak.

### Strengths and Limitations

The results of this study indicate that the Internet could be used for investigating epidemiological and clinical characteristics of such infectious disease outbreaks as the H7N9 outbreak. Different from many Web-based biosecurity intelligence systems such as HealthMap, MedISys, and BioCaster, our investigation is aimed to provide a comprehensive and more detailed investigation of a disease outbreak. Most of these intelligence systems are established merely for alerting of health threats by providing only a summary number of cases over time without detailed information important and necessary for understanding and controlling an infectious disease outbreak.

Compared with traditional epidemiological investigations of disease outbreaks, Internet-based investigations have a few important advantages. First, Internet-based data can be quickly analyzed and reported and initial results can be useful in facilitating efficient contingency planning in particular in the early stage of an epidemic. Second, the coverage of the data has no boundary and can be national or international and thus can provide an overall picture of the problem any local and regional data cannot. Third, as publically available Internet data are open to any potentially interested people worldwide with the skills to do the analyses. This can greatly facilitate the timely analysis and update of an outbreak so that rapid and appropriate action can be taken for its control. Fourth, early initial Internet data and their analyses may greatly help alert and pressure governments and authorities to facilitate timely publication of all the data to the general public, ideally through the Internet, which can facilitate timely action against the outbreak by preventing intents of withholding data for other reasons, such as motivations resulting from political parallelism or the benefits from publishing a high impact factor journal paper. Fifth, Internet data can be easily complemented, counterchecked, and corrected by others who have access to all or some of the original data from different sources. Finally, if an epidemic persists, in particular when it involves only a few sporadic cases, data on newly confirmed cases could be collected through the Internet for analysis and updating.

The major problem of Internet-based investigations is incomplete data. For example, we had individual data on only 33 death cases by December 1, 2013, which resulted in a lower case fatality than that reported by the field study [17]. Exhaustive search of various data sources on the Internet may help reduce missing data. For summary results, such as case fatality, incomplete individual data can often be overcome by using the aggregate data that are normally more conveniently available on the Internet.

Incomplete data may become particularly severe if the epidemic persists for a lengthy period of time or at the late stage as the event is dying out and gradually moving out of the public’s attention. In our case, for example, even aggregate data on the number of deaths from December 1, 2013 to February 10, 2014 as the early part of the second outbreak were not traceable on the Internet. We estimated the case fatality only based on limited data on individual cases identified. The estimate was lower than that reported in Li’s study [17] for the outbreak period before December 1, 2014, but similar to that reported in Wikipedia [23], which showed that as of March 1, 2014, there were 375 confirmed cases of H7N9 and 80 people had died resulting in a case fatality rate of 21.3%. Maybe the case fatality rate for H7N9 was indeed lower in the second outbreak between the late 2013 and the early 2014 than that in the early 2013. Official aggregate data are yet needed to confirm this result.

Incomplete data are more likely to occur on details of individual cases than the cases themselves. We found clinical characteristics were often missing for individual cases. Furthermore, our epidemic curve was similar in the shape, time period, peak date, and total number of cases to those reported in Li’s study before December 1, 2013. However, between December 1, 2013 and February 10, 2014, 196 cases were identified for the second outbreak but the date of illness onset was available only for 30 patients, which prevented us from describing reliably the epidemic curve for the second outbreak. This indicates that Internet-based methods may be, for the time being, more suitable for investigating the early stage of sudden and short-lasting events of public concern.

### Conclusions

This study presented a new outbreak of H7N9 infection in China by using publicly accessible data on the Internet. The results of the study agreed in most epidemiological and clinical characteristics with those from an authoritative conventional epidemiologic study [17]. Internet-based investigations appear particularly useful for sudden, emergent, relatively short-lasting issues or events of great public concern that involve a relatively small number of cases and for which relevant data can be conveniently uploaded and updated on the Internet. Such investigations can be conducted by any interested people who have the skills to do the analyses so that the issue can be publicized more quickly and updated for quicker and better understanding and controlling of the problem. It will greatly facilitate such investigations if governments, authorities, organizations, or individuals can consciously and systematically put relevant data on the Internet and make them publicly accessible for the greater good of the public.

## Multimedia Appendix 1

Individual data for patients with confirmed H7N9 infection from the Internet.

## Abbreviations

ARDS: acute respiratory distress syndrome
CDC: Center for Disease Control and Prevention
ICU: intensive care unit
WHO: World Health Organization
SARS: severe acute respiratory syndrome
